# Combined ADC and T_2_ Mapping in the Prostate Using a 3D Reduced FOV Sequence

**DOI:** 10.1002/mrm.70510

**Published:** 2026-07-14

**Authors:** Yannik Ott, Sarah McElroy, Raphael Tomi‐Tricot, Carolyn Horst, Patricia A. Gutierrez, Rahul Khamar, Omar Darwish, Vicky Goh, Radhouene Neji

**Affiliations:** ^1^ Research Department of Imaging Physics and Engineering School of Biomedical Engineering and Imaging Sciences, King's College London London UK; ^2^ Research and Clinical Translation, Magnetic Resonance Siemens Healthineers AG Erlangen Germany; ^3^ MR Research Collaborations Siemens Healthcare Ltd Camberley UK; ^4^ Siemens Healthcare SAS Courbevoie France; ^5^ Research Department of Cancer Imaging School of Biomedical Engineering and Imaging Sciences, King's College London London UK; ^6^ Department of Radiology Guy's and St Thomas' NHS Foundation Trust London UK; ^7^ Department of Radiology Centre Hospitalier Dunkerque Dunkirk France

**Keywords:** diffusion‐prepared imaging, distortion‐free diffusion, prostate MRI, reduced FOV, T_2_ mapping

## Abstract

**Purpose:**

To develop a 3D reduced FOV sequence for combined ADC and T_2_ mapping in the prostate in a single scan.

**Methods:**

A 3D ADC and T_2_ mapping reduced FOV acquisition is enabled using T_2_ and diffusion preparation modules with slab‐selective tip‐down pulses and magnitude stabilizer gradients. Imaging shots are followed by 2D phase navigators for phase correction of diffusion‐prepared shots and dummy RF pulses for T_2_‐prepared shots, as well as a constant delay to maintain the steady state of the longitudinal magnetization. Mono‐exponential fitting is used for ADC and T_2_ mapping. T_2_ mapping accuracy was assessed in a phantom against a single‐echo spin‐echo reference. In vivo, the sequence was compared in the prostate of 11 healthy volunteers against a multi‐echo spin‐echo acquisition, as well as standard and reduced FOV (rFOV) single‐shot EPI (ssEPI).

**Results:**

In the phantom experiments, T_2_ values were not significantly different from the reference single‐echo spin‐echo measurements. ADC values in vivo were not significantly different from those obtained using ssEPI. T_2_ values in vivo showed a mean bias of −21.7 ms compared to those obtained using multi‐echo spin‐echo with the first echo discarded in the fitting. The proposed sequence achieved similar overall image quality scores to those of ssEPI and rFOV ssEPI, improved perceived distortion scores, but lower perceived SNR scores.

**Conclusion:**

The proposed approach enables 3D reduced FOV, single‐scan ADC, and T_2_ mapping with mono‐exponential fitting in the prostate.

## 1 Introduction

Prostate cancer accounts for 15% of cancers worldwide and is the most common cancer amongst men in 112 countries [1]. MRI plays an essential role in the diagnosis of prostate cancer: it helps detect clinically significant cancer [2] and reduces the overdiagnosis of clinically insignificant cancer [3]. PI‐RADS (Prostate Imaging—Reporting and Data System) v2.1, the standard for reporting multiparametric prostate MRI worldwide, is based predominantly on qualitative visual assessment of two primary contrasts, namely diffusion‐weighted (DWI) and T_2_‐weighted MRI, to detect lesions and determine the likelihood of clinically significant cancer (PI‐RADS 4 or 5) [4]; though dynamic contrast‐enhanced (DCE) MRI has a role in further characterizing indeterminate PI‐RADS 3 lesions following DWI and T_2_‐weighted MRI [5, 6]. A single sequence combining DWI and T_2_‐weighted information, and leveraging additional quantitative ADC [7, 8] and T_2_ [8, 9, 10] relaxation values, has the potential to improve prostate lesion characterization and diagnostic performance for clinically significant prostate cancer, reducing the need to proceed to DCE MRI. Moreover, a robust, fast (˜10 min) joint diffusion and T_2_ quantitative sequence could be beneficial in the context of prostate cancer screening [11, 12], since early detection of clinically significant cancer and improved lesion characterization could lead to higher survival rates while reducing overdiagnosis of clinically insignificant cancer. Nevertheless, a key problem is that standard clinical EPI‐based DWI sequences may be affected by geometric distortions [13] so the quantitative maps obtained from such a sequence may suffer from misalignment, hence compromising the concept of joint quantification with spatial fidelity. Field map‐based distortion correction techniques [14, 15] can mitigate geometric distortions in standard EPI scans by acquiring an additional scan with a reverse phase encoding direction, but residual distortions persist, particularly in regions with strong magnetic susceptibility gradients [16, 17].

In this context, diffusion preparation [18] modules have been proposed to enable distortion‐free multi‐shot DWI by allowing for flexible imaging readouts [19, 20, 21, 22, 23, 24, 25]. The major challenge associated with this technique is spatially varying magnitude errors caused by phase errors due to motion and eddy currents before the tip‐up pulse [20]. To address these challenges, magnitude stabilizer gradients [26, 27, 28, 29, 30] combined with inter‐shot phase correction were proposed. Diffusion preparation modules are particularly interesting in the context of joint ADC and T_2_ quantification because they are essentially an extension of T_2_ preparation modules, which are widely used for T_2_ mapping [31, 32, 33].

Distortion‐free sequences for joint quantification of ADC and T_2_ relaxation times include magnetic resonance fingerprinting [34, 35] (MRF), magnetic resonance multitasking [27, 36], and RF‐modulated phase‐based diffusion [37] (PBD). However, MRF requires simulating a new dictionary for each new protocol adaptation that leads to a change of signal time course, while the low‐rank tensor reconstruction used in MR multitasking to enable high acceleration factors and the reconstruction required to solve the nonlinear inverse problem defined by the complex signal model of RF‐modulated PBD lead to long reconstruction times. This is also a limitation of echo planar time‐resolved imaging [38] which enables distortion‐free diffusion [39, 40, 41] and T_2_ mapping [42] but requires complex reconstruction algorithms due to high acceleration in k_y_‐t space.

3D T_2_ mapping in the prostate based on T_2_ preparations, Cartesian spiral profile ordering, and dictionary matching has been previously proposed [31]. Furthermore, 3D distortion‐free diffusion‐prepared imaging of the prostate has been proposed using phase‐cycling of the phase of the tip up pulse in the diffusion preparation and dictionary matching [43] or using reduced FOV acquisitions [28]. However, these techniques do not enable a combined T_2_ and ADC quantification approach, and dictionary generation and matching are computationally expensive and dependent on sequence parameters.

Imaging small organs such as the prostate favors the application of reduced FOV [44, 45, 46, 47] techniques, where the number of phase encoding lines per shot is reduced to naturally accelerate the acquisition without a substantial increase in the computational complexity of the reconstruction. Moreover, the use of magnitude stabilizers is typically combined with stimulated echo [48] (STE) sequences, where shorter shots due to a reduced FOV mitigate PSF‐related blurring [49] due to T_1_ decay. Furthermore, a reduced FOV approach in prostate MRI enables the use of frequency encoding along the anterior–posterior direction to mitigate pelvic wall motion artifacts at no additional scan time cost.

In this work, we extend a previously proposed 3D reduced FOV diffusion‐prepared sequence [30] with additional reduced FOV T_2_ preparation modules so that natively co‐registered distortion‐free 3D prostate ADC and T_2_ maps can be obtained. The sequence design maintains a steady state which is independent of the signal weighting by either diffusion or T_2_ preparation, enabling ADC and T_2_ estimation by simple mono‐exponential fitting. Therefore, the fitting procedure does not require protocol‐specific dictionary generation followed by dictionary matching. The proposed approach was validated at 3 T using phantom experiments and prostate scans in vivo in healthy volunteers.

## 2 Methods

### 2.1 Data Acquisition

This work builds on the method previously proposed by McElroy et al. [30], which introduced a reduced FOV 3D gradient echo sequence for diffusion‐prepared imaging based on turbo‐STEAM [50, 51] and is similar in its design to the multi‐tasking sequence introduced by Ma et al. [27] for joint T_1_, T_2_, and ADC mapping. The sequence proposed by McElroy et al. [30] is extended by including additional T_2_‐preparation (T2P) modules with varying T_2_ preparation times t_T2P_ to enable joint T_2_ and ADC mapping within a single 3D reduced FOV acquisition.

**FIGURE 1:**
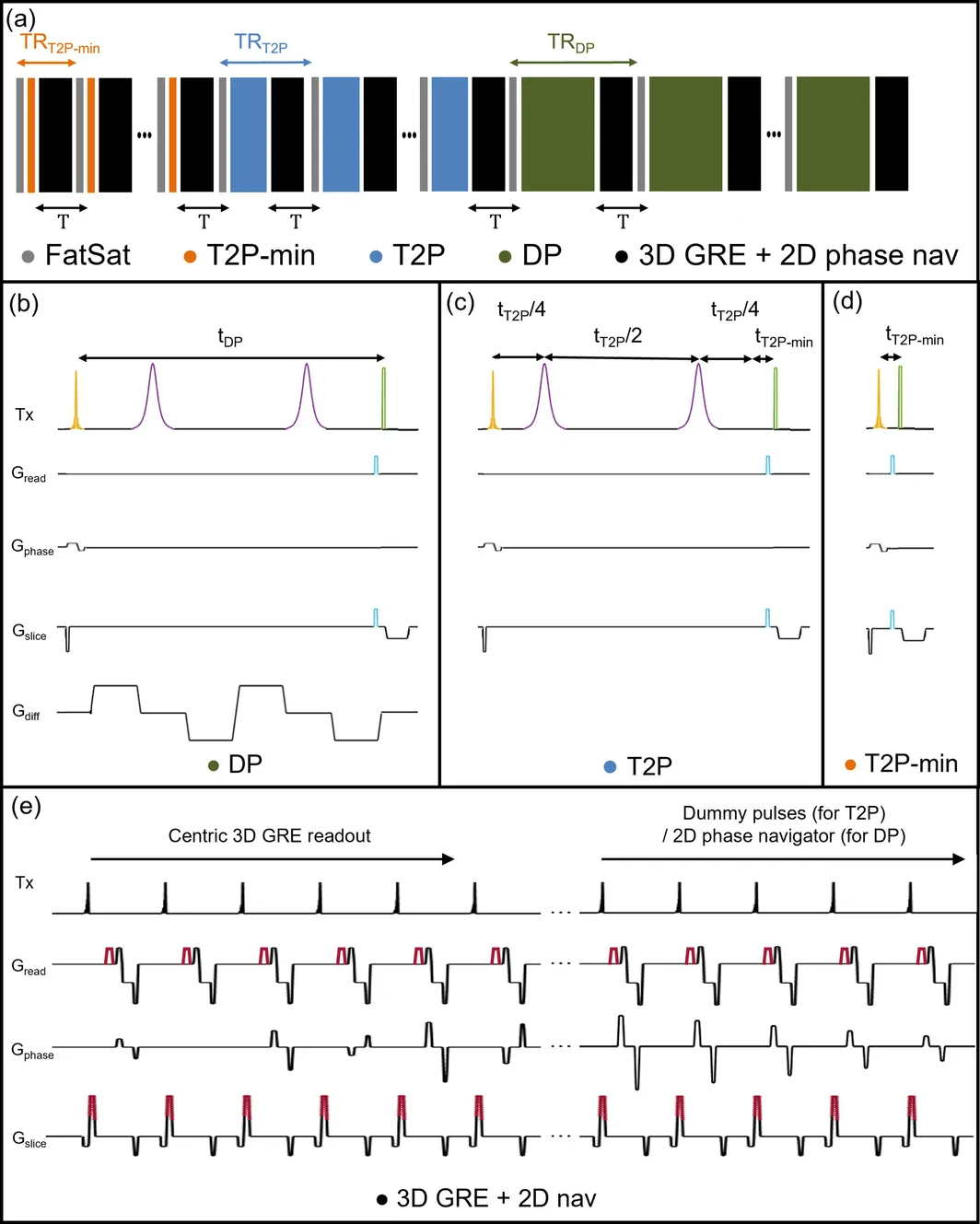
Overview of the proposed multi‐shot sequence (subfigure a). Shots start with a chemical shift‐selective fat saturation pulse (gray) followed by either diffusion preparation (green, subfigure b), T2 preparation (blue, subfigure c), or a reduced version of T2 preparation (orange, subfigure d) and finally a 3D gradient echo (GRE) imaging readout and a 2D phase navigator/dummy RF pulses (black, subfigure e). T2P modules start with a slab‐selective (in the phase encoding direction) tip‐down pulse (yellow), followed by twin adiabatic refocusing pulses (purple), magnitude stabilizer dephasing gradients (light blue), and a tip‐up pulse (light green) followed by a spoiling gradient. Magnitude stabilizer rewinder gradients are applied in the 3D GRE readout (red, subfigure e). The reduced version T2P‐min consists of tip‐down and tip‐up pulses and the magnitude stabilizer gradients only to maintain the reduced FOV approach (subfigure d). DP modules are T2P modules with additional diffusion‐weighting gradients. The delay of the T2P‐min module is also added to all other modules (subfigure c). The time T between every tip‐down and subsequent tip‐up pulse remains constant throughout the acquisition to ensure a constant steady‐state longitudinal magnetization, which means that different T2 or diffusion preparations may have different shot‐to‐shot repetition times (subfigure a).

The proposed multi‐shot sequence is shown in Figure 1. Each shot acquires all the phase encoding lines for one partition of the imaged 3D volume. Each shot starts with a chemical shift‐selective fat saturation pulse [52], followed by a magnetization‐preparation module that introduces either diffusion or T_2_ weighting to the longitudinal magnetization.

The T2P module (Figure 1c) is similar in its design to the diffusion‐preparation (DP) module (Figure 1b) previously described [28, 30]: a tip‐down pulse is applied simultaneously with a slab‐selective gradient in the phase encoding direction to restrict the excitation to a reduced FOV and this is combined with twin adiabatic refocusing pulses [53], magnitude stabilizer gradients applied in both the slice and the readout directions, and a nonselective tip‐up pulse. The preparation module is followed by a spoiling gradient with empirically adjusted duration (6.6 ms) and amplitude (20 mT/m) to spoil remaining transverse magnetization, particularly the unwanted spin‐echo pathway generated by the tip‐down and tip‐up pulses.

Following magnetization preparation, the imaging data are acquired using a centric gradient echo readout, combined with magnitude stabilizer rewinders. A 2D phase navigator [54] without partition‐encoding is acquired following each diffusion‐prepared shot to measure shot‐specific phase variations. The following is ensured so that a steady state for the longitudinal magnetization prior to each tip down pulse is maintained (after one dummy shot): the RF pulses of the 2D phase navigator are also applied as dummy RF pulses following the T_2_‐prepared imaging shots, and the time Ͳ between the tip‐up pulse and the subsequent tip down pulse is kept constant during the whole acquisition, which leads to a shot‐to‐shot repetition time that is variable with respect to the duration of the preparation module (Figure 1a).

Conventional magnetization‐prepared T_2_ mapping techniques acquire one dataset without T_2_ preparation (*t*_T2P_ = 0 ms). However, in order to maintain a reduced FOV acquisition, the proposed sequence instead includes a minimal T2P module (T2P‐min) consisting of a tip‐down pulse, magnitude stabilizer gradients, and a tip‐up pulse (Figure 1d). This introduces a small degree of T_2_*‐weighting to the prepared magnetization due to the delay *t*_T2P‐min_ required for this reduced preparation module (or T_2_‐weighting in case the echo time of the gradient echo readout is chosen to be equal to *t*_T2P‐min_, due to stimulated echo refocusing of B_0_ inhomogeneities). The delay *t*_T2P‐min_ is the sum of half the durations of the tip‐up and tip‐down pulses, and the durations of the slab refocusing and magnitude stabilizer gradients, which in our implementation corresponds to *t*_T2P‐min_ = 4.15 ms. To ensure that the delay *t*_T2P‐min_ results in a constant scaling factor across all acquired T_2_‐prepared signals without affecting the T_2_ fitting, this duration is added to the other T2P modules between the second refocusing pulse and the tip‐up pulse, as shown in Figure 1c.

The sequence successively acquires magnetization‐prepared data starting with T_2_‐prepared data with increasing T2P durations, followed by diffusion‐prepared data in the following acquisition order: *b*‐values, diffusion directions, shots, and averages.

### 2.2 Image Reconstruction and ADC/T2 Fitting

**FIGURE 2:**
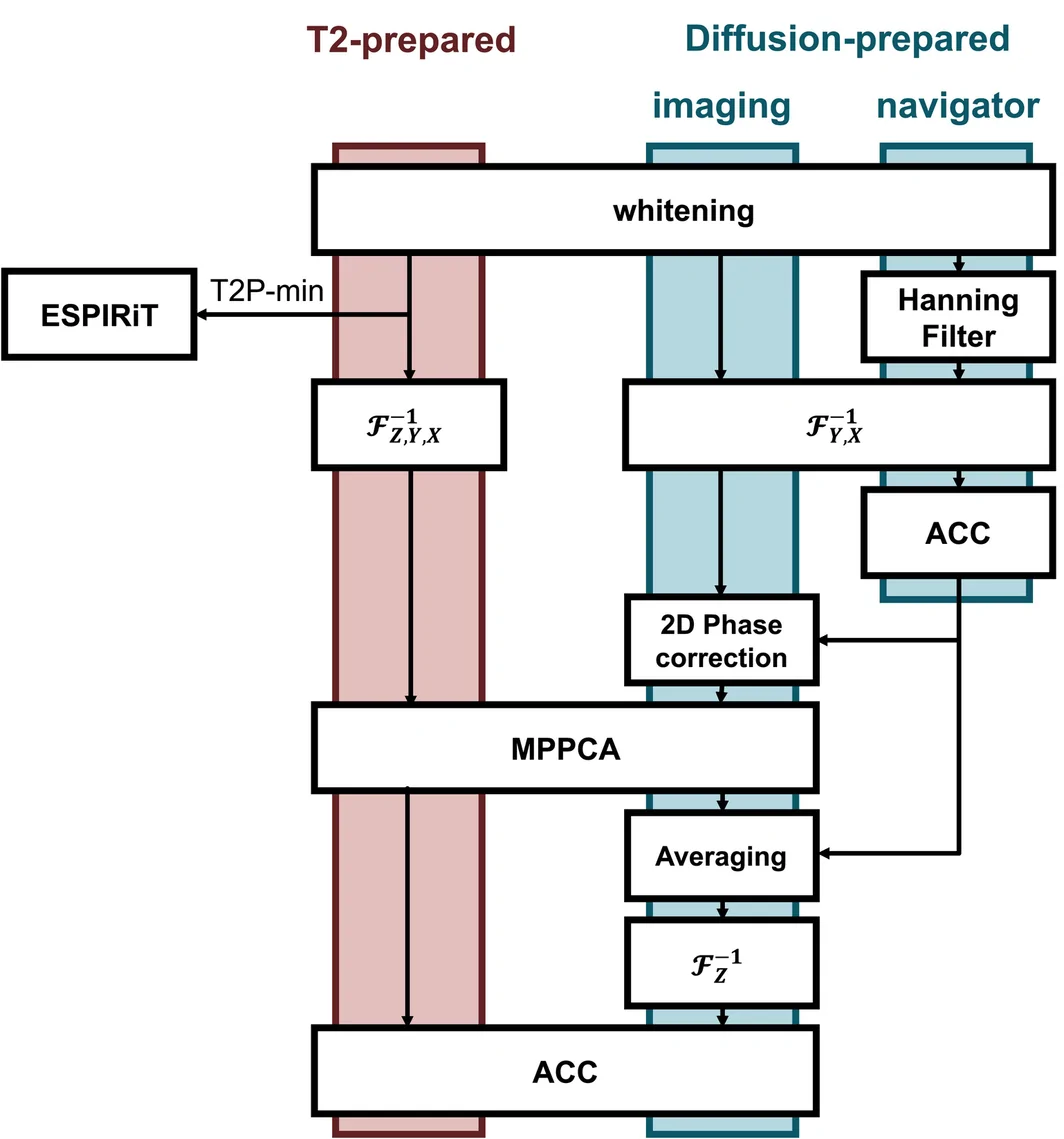
Reconstruction pipeline of images with T2 preparation (red) and diffusion preparation (blue). First, ESPIRiT is used to estimate 3D coil sensitivity maps based on the T2P‐min dataset. T2P datasets are Fourier transformed and adaptive coil combination (ACC) is used to obtain the final T2P images. A Hanning filter is applied along the phase encoding direction of the 2D phase navigator. Then, the navigator is Fourier transformed and coils are combined using a projection of the 3D coil sensitivities along the partition dimension. Subsequently, the diffusion‐prepared data is Fourier transformed along the in‐plane dimensions and phase‐correction and magnitude‐based averaging are performed with respect to the coil‐combined navigator. Finally, the phase‐corrected diffusion‐prepared data is Fourier transformed along the partition dimension and ACC yields the DP images. Prior to averaging, multi‐contrast MPPCA denoising is performed on diffusion‐ and T2‐prepared imaging data.

Figure 2 depicts the reconstruction pipeline. First, coil sensitivities are estimated using the ESPIRiT algorithm [55] based on the T2P‐min acquisition. T_2_‐prepared images are reconstructed by applying an inverse Fourier transformation to the corresponding k‐space data followed by adaptive coil combination (ACC). For diffusion‐prepared shots, a Hanning filter is applied along the phase‐encoding direction of each phase navigator to avoid Gibbs ringing artifacts. Imaging and navigator data are inverse Fourier transformed along the in‐plane dimensions. Furthermore, 2D coil sensitivity maps, obtained by projecting along the shot/partition dimension, are used for coil combination of the 2D phase navigators. Inter‐shot phase inconsistencies of diffusion‐prepared shots are removed by subtracting the coil‐combined shot‐specific navigator phase from each shot. Multiple averages for the diffusion‐prepared data are combined using weighted spatially‐adaptive averaging based on normalized squared navigator magnitude signals as combination weights to lower the contribution from diffusion‐prepared shots with strong intravoxel dephasing. Equation (1) illustrates the combined phase correction and magnitude‐based averaging of diffusion‐prepared data to obtain the phase corrected image data $\hat{s}$ from its phase corrupted version s and corresponding navigator data n.

(1) $${\hat{s}}_{s,c}=\sum_{a=0}^{N_{a}}\frac{{\left|n_{a,s}\right|}^{2}}{\sum_{a=0}^{N_{a}}{\left|n_{a,s}\right|}^{2}}\;\left|s_{a,s,c}\right|e^{j\left(∠s_{a,s,c}-∠n_{a,s}\right)}$$

Here, a, c, s denote the average, coil, and shot indices, respectively and $N_{a}$ is the number of averages. Note that for the phase correction, imaging and navigator data are inverse Fourier transformed along the y and x dimension only since the phase of each shot must be corrected using the non‐partition encoded navigator prior to reconstruction. Subsequently, an inverse Fourier transform is applied along the partition dimension and ACC is used to obtain the final diffusion‐prepared images. Please note, prior to averaging, MPPCA denoising [56] is applied jointly to all diffusion‐ and T_2_‐prepared imaging data as proposed in multi‐contrast MPPCA [57]. Finally, the N4 algorithm [58] was used to estimate the receiver coil bias field from the T2P‐min image, to jointly correct intensity inhomogeneities of all images.

A mono‐exponential fitting model is used to compute ADC and T_2_ relaxation values from the diffusion‐prepared data and the T_2_‐prepared image data, respectively.

Image reconstruction was implemented offline in Python and performed on a NVIDIA RTX A4000 GPU, while the MPPCA denoising [56] and bias field correction were based on a CPU‐only implementation.

### 2.3 Experiments

The proposed sequence was compared against standard single‐shot EPI (ssEPI) and a reduced FOV single‐shot EPI [59, 60] (rFOV ssEPI) vendor implementation as reference sequences for diffusion imaging. The T_2_ maps of the proposed approach were compared against those obtained using a multi‐echo spin echo (meSE) acquisition with two different T_2_ fitting procedures. First, T_2_ maps were calculated using mono‐exponential fitting of all acquired echo images. However, meSE sequences suffer from contamination of the spin‐echo pathway with stimulated echo contributions due to imperfect refocusing and slice profiles, which may cause the second acquired echo to be higher than the first [61, 62]. Therefore, an additional T_2_ map was obtained by performing a T_2_ fitting excluding the first echo image [63]. Table 1 summarizes the sequence parameters. All acquisitions were measured using a slice thickness of 4 mm, phase‐encoding direction right → left, and in transverse orientation.

**TABLE 1:** Sequence parameters for in vivo studies. Please note that for the proposed sequence T2 and diffusion preparation durations are the main factors behind the amount of T2 weighting while it is TE for all the spin echo sequences used in this study (ssEPI, rFOV ssEPI, meSE, and TSE).

	Proposed	rFOV ssEPI	ssEPI	meSE	TSE
TE (spin echo) [ms]	—	106	87	23, 47, 70, 93, 117, 140	108
TE (gradient echo) [ms]	3	—			
TR [ms]	1836–1976*	4000		2500	6010
Effective echo spacing [ms]	5.13	0.36		—	—
In‐plane resolution [mm × mm]	2.34 × 2.34				0.85 × 0.85
FOV (read × phase) [mm × mm]	300 × 113	300 × 159	300 × 300		
Phase oversampling [%]	10	20		70	100
Receiver bandwidth [Hz/Px]	500	1562		250	200
Acceleration	None		GRAPPA 2	GRAPPA 3	GRAPPA 3
Excitation flip angle [°]	10	90			
Fat suppression	FatSat	FatSat w/gradient reversal		FatSat	None
*b* value (averages) [×10^−3^ mm^2^/s]	0 (1), 800 (3–4)	0 (1), 800 (25)		—	—
Acquisition time [min:s]	10:34 ± 00:43 (08:37–11:56)	05:14	05:18	04:07	3:14

Abbreviations: FatSat, fat saturation; meSE, multi‐echo spin‐echo; rFOV, reduced FOV; ssEPI, single‐shot EPI; TSE, turbo spin‐echo.

* Variable for different preparation modules.

In all experiments, statistical significance between two groups of values were assessed using the Wilcoxon signed rank test after ensuring that the groups were nonnormal distributed using the Shapiro–Wilk test. For both tests a significance level of α=0.05 was used.

#### 2.3.1 Phantom Study

To evaluate the accuracy of the proposed T_2_ mapping, our approach and both meSE T_2_ maps were compared against the gold standard single‐echo spin‐echo (seSE) sequence with acquisitions at different echo times. The T1MES standardized T_1_ and T_2_ phantom with vials of different T_2_ and T_1_ relaxation times was used [64]. Sequence parameters are listed in Table S1. All images were acquired at an in‐plane resolution of 2.34 × 2.34 mm^2^. For the proposed sequence, T_2_‐preparation durations of 0, 40, and 80 ms were used. The number of shots for the proposed sequence was 48. All phantom experiments were performed at 3 T (Biograph mMR, Siemens Healthineers, Forchheim, Germany).

#### 2.3.2 In Vivo Study

11 healthy volunteers (male, 67 ± 15 years, BMI = 26.89 ± 3.26 kg/m^2^) were recruited prospectively and scanned on a 3 T MRI scanner (MAGNETOM Prisma, Siemens Healthineers, Forchheim, Germany). All volunteers received an informed consent discussion and provided written consent for the further use and processing of their data. MR measurements were conducted in accordance with the Declaration of Helsinki. In addition to the proposed, ssEPI, rFOV ssEPI, and meSE sequences, a standard 2D T_2_‐weighted turbo spin echo (TSE) acquisition was acquired for anatomical reference. Sequence parameters are listed in Table 1. To ensure full prostate coverage, 8–14 slices were obtained. The proposed sequence was acquired with 100% slice oversampling to avoid fold‐over in the partition encoding direction due to imperfect slab excitation profile, resulting in 16–28 shots. Diffusion preparation duration was 60 ms. T_2_‐preparation durations were 0, 100, 140 ms, respectively. Here, a b0 image was acquired and included in both ADC and T_2_ fitting, that is, the ADC value was obtained as ADC = ln(S_0_/S_b_)/b, while the T_2_ map was generated by incorporating the b0 signal as reference point with a T_2_‐prep duration of 60 ms into the mono‐exponential model.

A reader study was performed to qualitatively compare the b800 trace‐weighted images of ssEPI, rFOV ssEPI, and the proposed method. A radiologist with > 20 years of experience rated overall image quality (1 = nondiagnostic, 2 = poor, 3 = fair, 4 = good, 5 = excellent), perceived SNR (1 = poor, 2 = fair, 3 = good, 4 = excellent), and perceived geometric distortions (1 = major distortion resulting in nondiagnostic image quality, 2 = major distortion but not limiting diagnosis, 3 = minor distortion but not limiting diagnosis, 4 = no distortion). To facilitate the assessment of geometric distortions, the TSE was provided with every rated image volume.

Subsequently, mean ADC and T_2_ values of the central–/transitional zone and peripheral zone for the proposed and the reference sequences were compared within manually delineated contours. All contours were drawn on the anatomical TSE reference, reformatted, and refined to match the anatomy for each sequence. Bland–Altman and linear regression analysis were performed to compare the mean T_2_ and ADC values for each volunteer dataset. For ADC values, the ssEPI acquisition served as reference while the meSE approach excluding the first echo from the fitting provided the reference T_2_ values.

## 3 Results

### 3.1 Phantom Study

**FIGURE 3:**
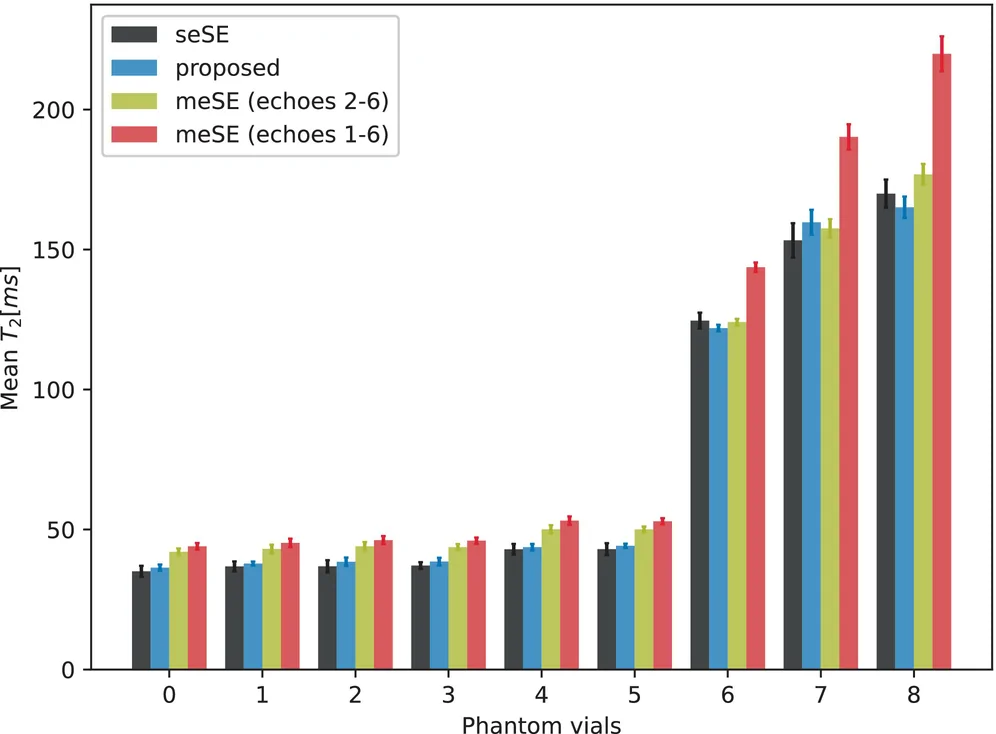
T2 mapping phantom study. Mean T2 values and standard deviation in each of the 9 phantom vials measured with a single‐echo spin echo (black), multi‐echo spin echo, where the first echo was excluded (green) or included (red) for the fitting, and the proposed sequence (blue).

Figure 3 depicts the mean T_2_ values for each individual phantom vial and sequence. In comparison to the reference T_2_ values provided by the seSE sequence, meSE (echoes 1–6), meSE (echoes 2–6), and the proposed approach yielded a mean bias of 23.68%, 12.59%, and 2.04%, respectively. There was no statistically significant difference in the obtained T_2_ values between the proposed approach and the seSE sequence (*p* = 0.43), while both T_2_ fitting procedures of the meSE acquisition differed significantly with respect to seSE (*p* ≤ 0.05). MeSE (echoes 1–6) shows higher bias with increasing T_2_ values.

### 3.2 In Vivo Study

**FIGURE 4:**
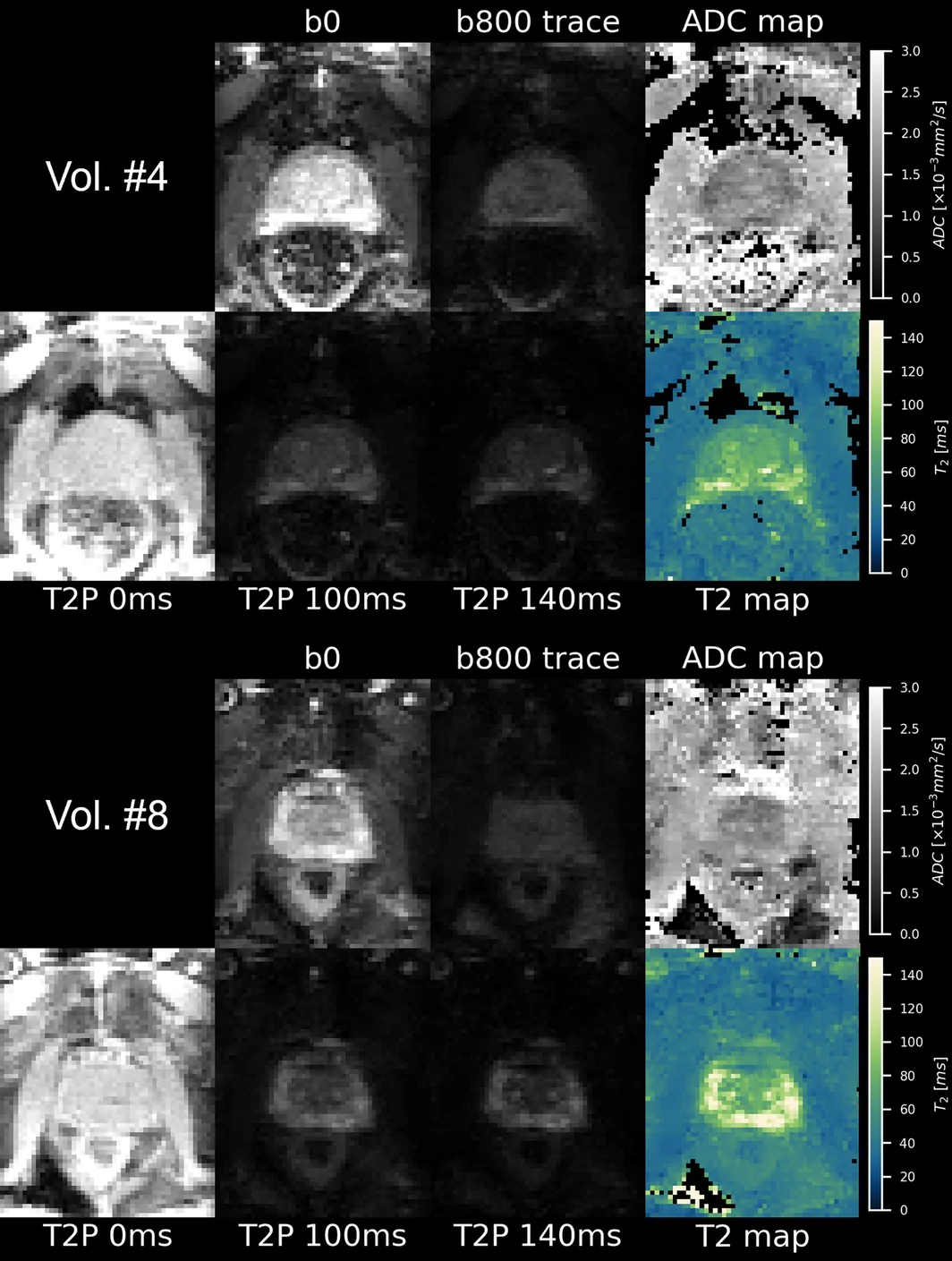
Examples of obtained diffusion‐ and T2‐prepared images as well as corresponding ADC and T2 maps for two representative volunteers.

**FIGURE 5:**
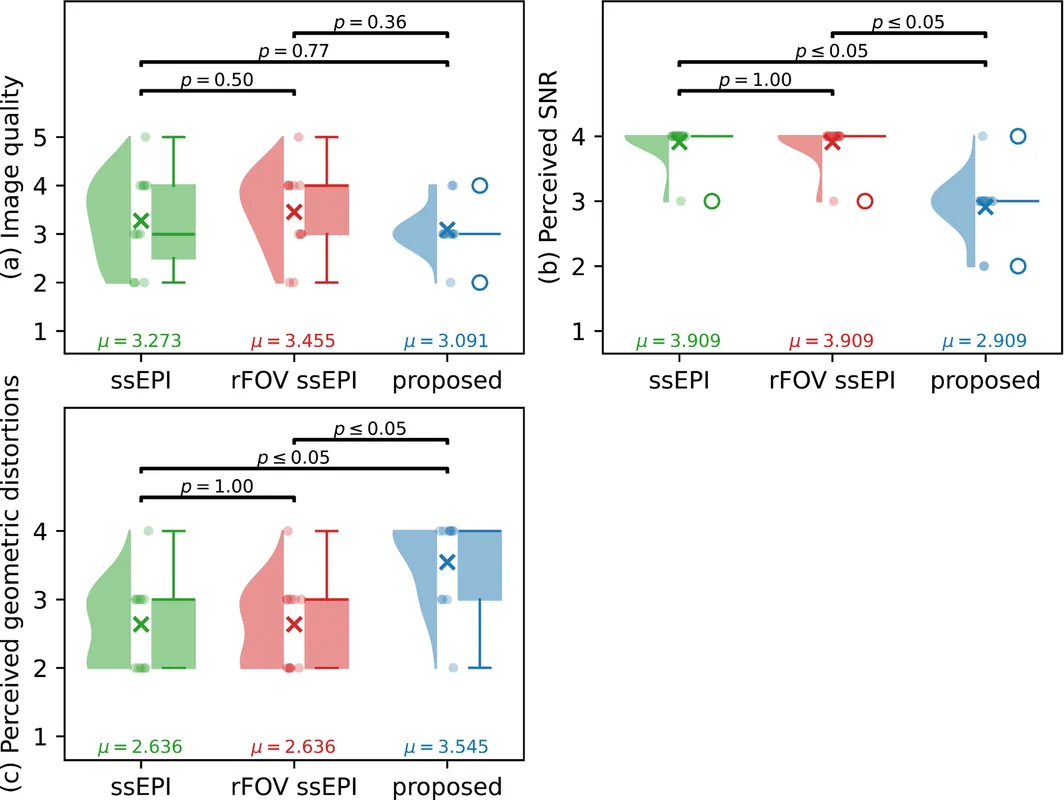
Results of the expert reader study comparing b800 trace‐weighted images of single‐shot EPI, reduced FOV single‐shot EPI, and the proposed technique. Violin and boxplot analysis of expert reader scores per subject with respect to image quality (a), perceived SNR (b), and perceived distortion (higher scores indicate less distortion) (c). Statistical significance was assessed using the Wilcoxon signed‐rank test (*α* = 0.05).

**FIGURE 6:**
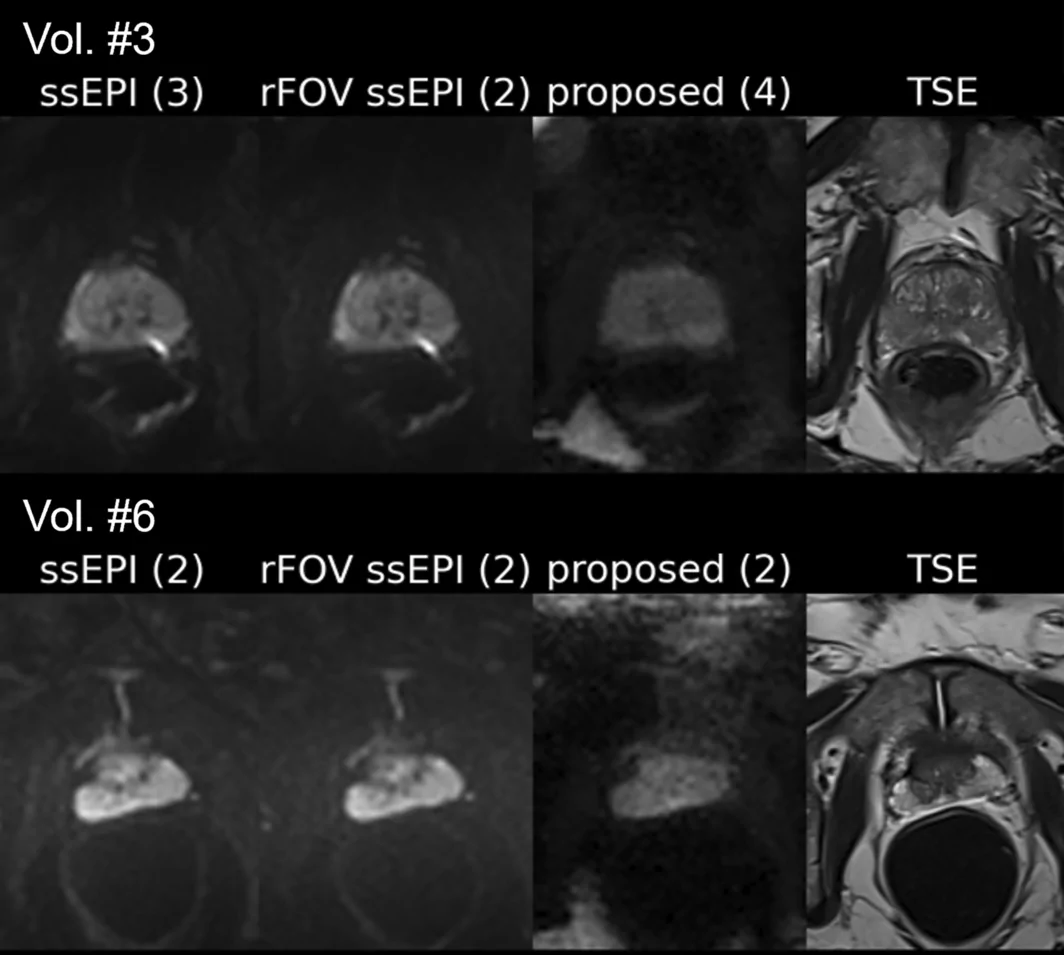
b800 trace‐weighted images of ssEPI, rFOV ssEPI, and the proposed sequence and T2‐weighted TSE for two volunteers. Image labels indicate the method, and the received perceived distortion score is provided between brackets.

Figure 4 shows representative images from two volunteer acquisitions. The mean overall image quality, perceived SNR, and perceived distortion scores obtained from the expert reader study for each method are shown in Figure 5. Regarding overall image quality, all methods performed comparably. The overall image quality scores of the proposed approach did not differ significantly from those of ssEPI (*p* = 0.77) and rFOV ssEPI (*p* = 0.36). Moreover, there was no statistically significant difference in the overall image quality scores of ssEPI and rFOV ssEPI (*p* = 0.50). With respect to perceived SNR, we observed no statistically significant difference between ssEPI and rFOV ssEPI (*p* = 1.00). However, the proposed approach yielded significantly lower perceived SNR scores than both EPI‐based techniques (*p* ≤ 0.05). The proposed approach achieved significantly higher perceived distortion scores than both EPI‐based sequences (*p* ≤ 0.05). There was no statistically significant difference found between the perceived distortion scores of ssEPI and rFOV ssEPI (*p* = 1.00). Figure 6 depicts b800 trace‐weighted images for two volunteers. For volunteer #3, perceived distortion scores were 3, 2, and 4, while for volunteer #6, the scores were 2, 2, and 2 for ssEPI, rFOV ssEPI, and the proposed sequence, respectively.

**FIGURE 7:**
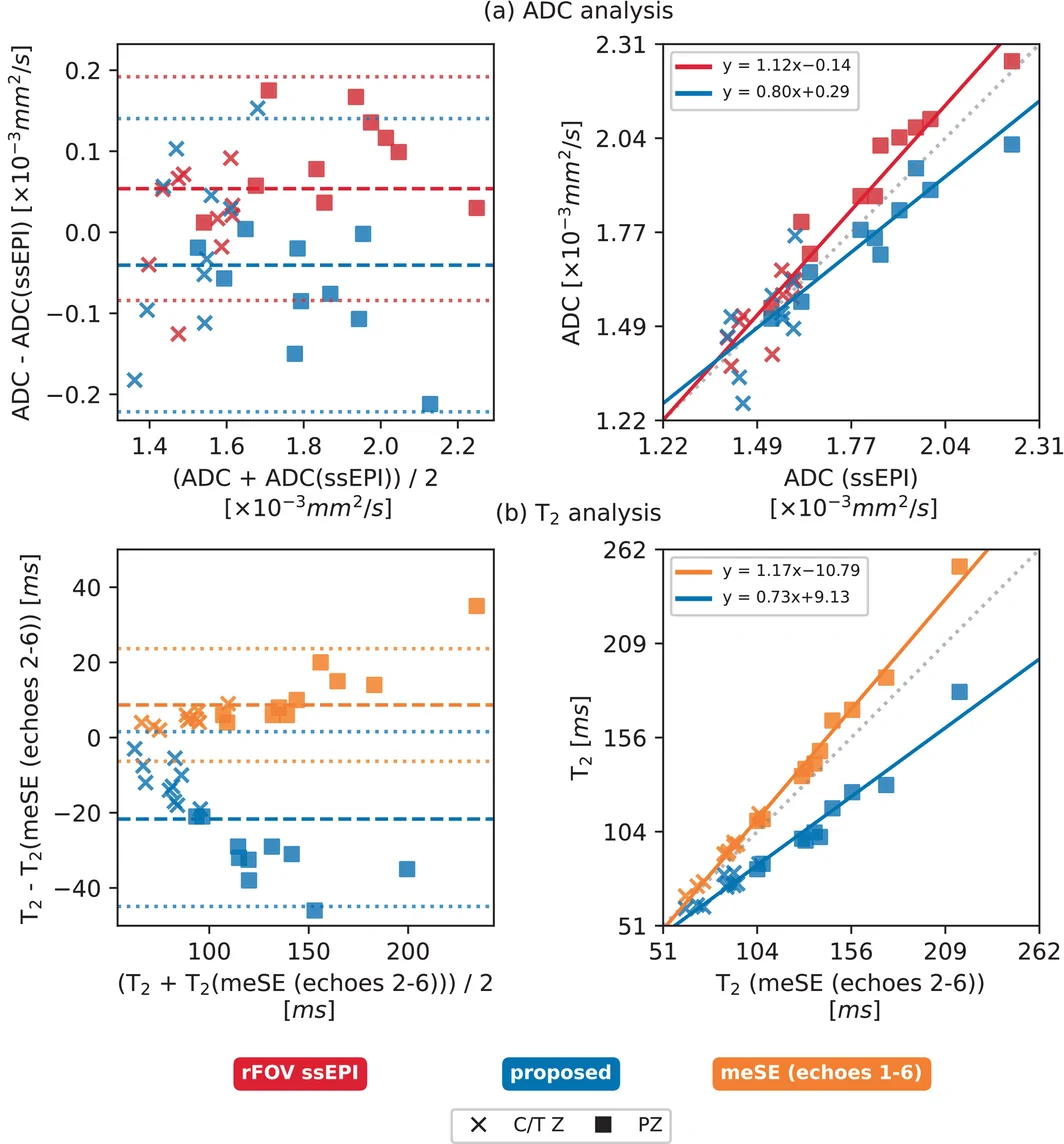
In vivo quantitative analysis. Bland–Altman (left) and linear regression (right) plots comparing mean ADC (a, top) values obtained using the proposed sequence and rFOV ssEPI sequence and mean T2 (b, bottom) values obtained using the proposed sequence and meSE (echoes 1–6) sequence in the central/transitional zone (cross) and peripheral zone (squares). For one volunteer no central/transitional zone was present and for another volunteer no peripheral zone was present, resulting in 20 data points. Reference values are obtained from ssEPI and meSE (echoes 2–6) for ADC and T2 values, respectively. Regarding ADC values, the proposed technique (blue) yielded a mean residual of −0.041 ± 0.181 [×10–3 mm^2^/s] (*p* = 0.06, R2 = 0.829) and rFOV ssEPI (red) a mean residual of 0.054 ± 0.138 [×10–3 mm^2^/s] (*p* ≤ 0.05, R2 = 0.935). Regarding T2 values, the proposed technique (blue) yielded a mean residual of −21.7 ± 23.2 [ms] (*p* ≤ 0.05, R2 = 0.993) and meSE (echoes 1–6) (orange) a mean residual of 8.7 ± 15.0 [ms] (*p* ≤ 0.05, R2 = 0.964).

**FIGURE 8:**
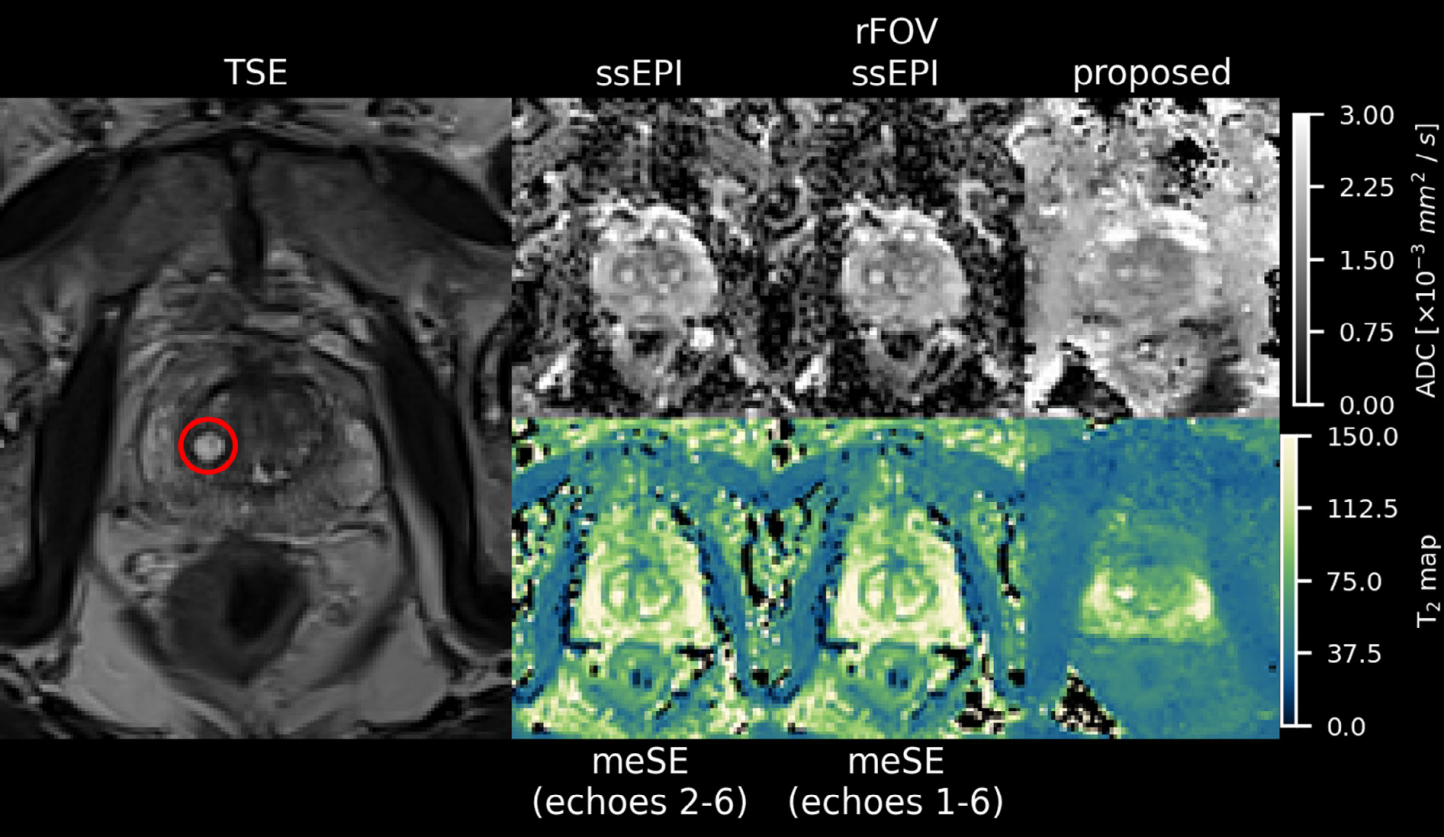
Comparison of ADC and T2 maps in one volunteer where a lesion was present in the central−/transitional zone with the T2‐weighted TSE acquisition as reference. Inside the lesion, an average ADC value of 1.92, 2.14, and 1.93 × 10–3 mm^2^/s was observed for the ssEPI, rFOV ssEPI, and the proposed sequence, respectively. The reference meSE (echoes 2–6), meSE (echoes 1–6), and the proposed sequence yielded a mean T2 value of 185, 241, and 127 ms, respectively.

Bland–Altman and linear regression plots comparing the mean ADC values in the prostate of all healthy subjects are shown in Figure 7a. The proposed approach yielded a statistically nonsignificant mean ADC underestimation of −0.041 × 10^−3^ mm^2^/s (*p* = 0.06) while the rFOV ssEPI approach overestimated mean ADC values by 0.054 × 10^−3^ mm^2^/s, and this underestimation was statistically significant (*p* ≤ 0.05). The 95% limits of agreement are wider for the proposed approach than for the rFOV ssEPI technique with 1.96*σ* of ±0.181 × 10^−3^ mm^2^/s and ±0.138 × 10^−3^ mm^2^/s, respectively. The linear regression coefficient of determination for the proposed and rFOV ssEPI were 0.829 and 0.935, respectively. Regarding T_2_ mapping, Bland–Altman plots and linear regression analysis are shown in Figure 7b. The meSE (echoes 1–6) approach yielded a mean overestimation of 8.7 ms and 95% limits of agreement of ±15.0 ms, while T_2_ values obtained by the proposed sequence resulted in a mean underestimation of 21.7 ms and 95% limits of agreement of ±23.2 ms. Both approaches yielded significantly different values from the reference method (*p* ≤ 0.05). T_2_ values that were fitted using meSE (echoes 1–6) demonstrate a trend of increasing overestimation for higher T_2_ values. Figure 8 shows quantitative maps as well as the TSE reference image of a slice containing a lesion. A mean ADC value of 1.92, 2.15, and 1.93 × 10^−3^ mm^2^/s was observed for the ssEPI, rFOV ssEPI, and the proposed sequence, respectively. Furthermore, meSE (echoes 2–6), meSE (echoes 1–6), and the proposed sequence yielded a mean T_2_ value of 185, 241, and 127 ms, respectively.

**FIGURE 9:**
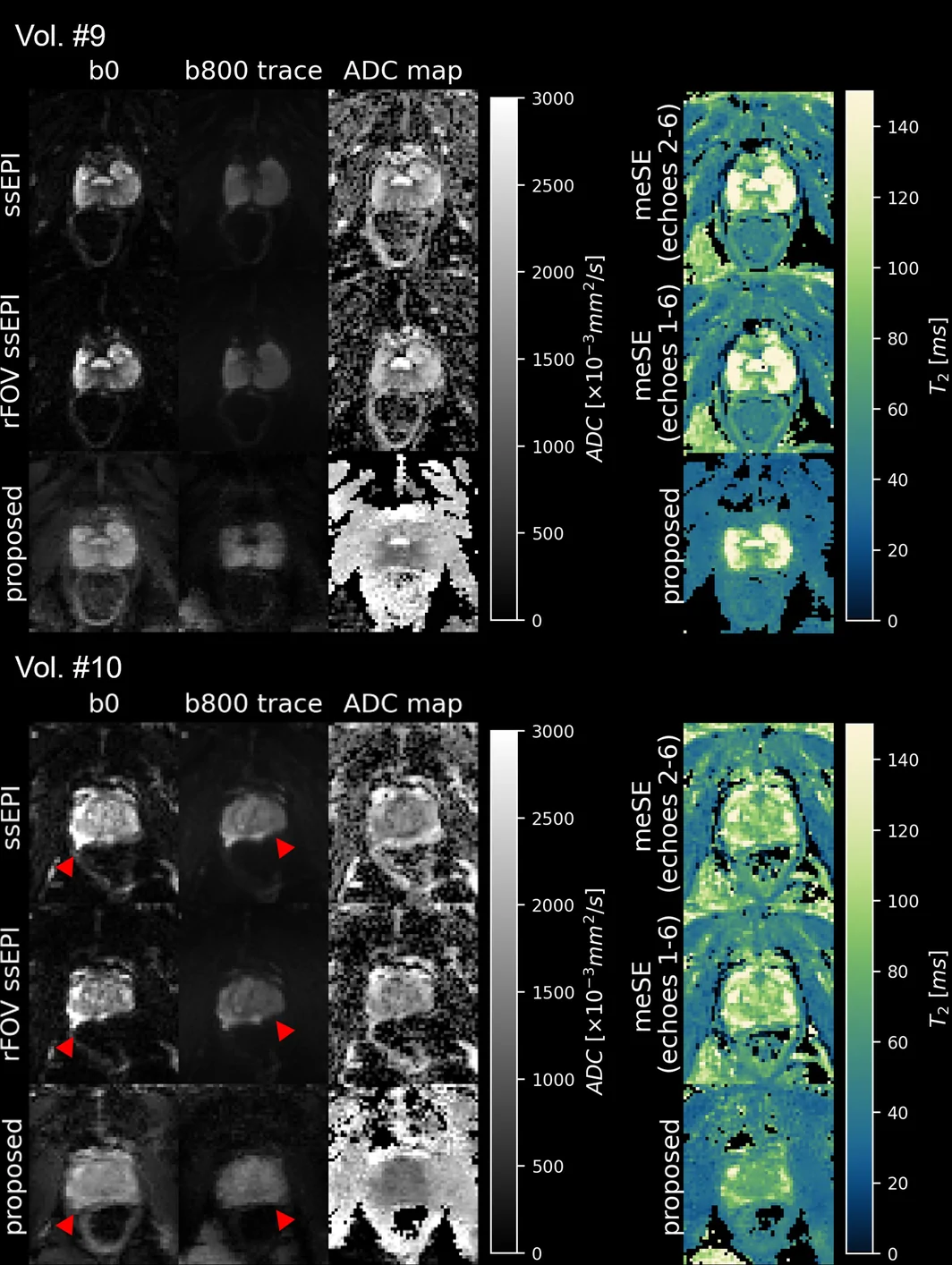
Comparison of diffusion‐weighted images, ADC‐ and T2‐ maps in two representative volunteers. Diffusion data were acquired using a single‐shot EPI, reduced FOV single‐shot EPI, and the proposed sequence. b800 images are displayed with increased brightness (×3). T2 maps of the proposed approach are compared against T2 maps acquired by two multi‐echo spin‐echo approaches: meSE which includes all echoes in the fitting (meSE (echoes 1–6)) and one which excludes the first echo from the fitting (meSE (echoes 2–6)). Red arrows point to regions where geometric distortions were observed in both ssEPI acquisition but not with the proposed sequence.

Figure 9 compares b0 and b800 trace‐weighted diffusion images and ADC maps of both EPI‐based acquisitions as well as the T_2_ maps of both meSE fitting approaches to the diffusion images, ADC, and T_2_ maps obtained using the proposed approach in two healthy volunteers. Regions can be observed where geometric distortions are present in both EPI‐based acquisitions but not in the proposed sequence.

An example of the result of the phase correction for all slices in the 3D volume is shown in Figure S1. Figure S2 compares b0 and b800 trace‐weighted diffusion images, including calculated b1400 images and ADC maps of both EPI‐based acquisitions and the proposed sequence.

On average, reconstruction and mono‐exponential fitting were completed in 1.43 s, the MPPCA denoising required 142.25 s, and the bias field correction required an additional 2.81 s.

## 4 Discussion

### 4.1 Interpretation of Results

We have proposed a 3D reduced FOV sequence to obtain natively co‐registered nondistorted ADC and T_2_ maps in the prostate. The sequence uses slab‐selective tip‐down pulses in the phase encoding direction for the T_2_ and diffusion preparation modules to achieve a reduced FOV. A steady state of the longitudinal magnetization is maintained prior to each tip‐down pulse to enable mono‐exponential fitting for ADC and T_2_ estimation.

In the T_2_ mapping phantom study, the T_2_ values obtained using the proposed approach were not statistically different from the gold‐standard single‐echo spin‐echo measurements. The meSE (echoes 1–6) approach showed a significant bias toward overestimation with a mean bias of 23.68%. However, excluding the first echo from the fitting (meSE (echoes 2–6)) lowered the bias to 12.59%.

Regarding in vivo quantification, there was no significant difference in the obtained prostate ADC values between the proposed approach and the reference ssEPI. However, the 95% limits of agreement were wider than those of the rFOV ssEPI technique, and this may reflect a lower precision in ADC quantification due to the lower SNR of the proposed approach. Both meSE (echoes 1–6) and the proposed method yielded significantly different T_2_ values in vivo from those obtained using meSE (echoes 2–6), with a mean bias of 8.7 and −21.7 ms, respectively. The observed in vivo biases can be explained by the lack of accuracy of the used meSE reference technique [65, 66]. It has been shown that even after discarding the first echo in the fitting, meSE still suffers from T_2_ overestimation due to imperfect refocusing pulses and stimulated echo contributions [65, 66]. A noticeable increase in over‐ and underestimation of T_2_ peripheral zone values can be observed for meSE and the proposed sequence, respectively. The higher deviation may be caused by longer T_2_ relaxation times, which is consistent with the findings of the phantom study, and with previous work showing increased bias of multi echo spin echo T_2_ mapping for longer T_2_ components (e.g., multi echo spin echo T_2_ mapping led to a ˜40 ms overestimation for T_2_ values of 145 ms [66]).

For our in vivo study, we chose to acquire with a maximum T_2_‐preparation duration of 140 ms, similarly to previous prostate T_2_ mapping studies [31, 67]. This value was chosen to increase sensitivity to long T_2_ components, while maintaining enough SNR. A T_2_ prep duration of 60 ms was selected for short components (72–85 ms for cancerous tissue [67, 68, 69, 70], and 76–97 ms for central and transition zones [31, 68, 70]) together with a T_2_‐prep duration of zero for its high SNR to stabilize the fit for these components. An additional T_2_‐prep duration of 100 ms was additionally acquired to account for intermediate T_2_ components. Prior studies [31, 67, 68, 69, 70] reported values from 97 to 170 ms for healthy peripheral zone tissue, which is closer to the observed values of our proposed sequence than both multi‐echo spin‐echo sequences.

In the expert reader study, the proposed approach performed comparably to standard and rFOV ssEPI techniques with respect to overall image quality and achieved significantly higher perceived distortion scores than both ssEPI sequences. However, the proposed approach had significantly lower perceived SNR scores compared to both ssEPI techniques. These results were expected since the proposed method achieves distortion‐free diffusion imaging with a gradient echo readout at the cost of the 50% SNR penalty associated with stimulated echoes [48].

### 4.2 Technical Aspects

A reduced FOV is beneficial in the context of 3D prostate quantitative imaging and is achieved using two mechanisms: the tip up pulse acts as a tip‐down pulse for the outer‐volume magnetization when the tip‐down is slice selective in the phase encoding direction, and the magnitude stabilizer rewinder gradients are crusher gradients for the outer volume magnetization [28, 30]. It enables shorter imaging shots and therefore mitigates the k‐space weighting introduced by T_1_ decay of stimulated echoes along the shot which can lead to blurring [49]. In addition, shorter imaging shots enable more T_1_ recovery period without RF excitation pulses, leading to a higher available steady state magnetization. Shorter imaging shots can be achieved using k‐space segmentation approaches [31, 43, 71], however, these would typically lead to a higher number of shots and longer acquisition times. For diffusion imaging with k‐space segmentation strategies, it is not guaranteed that the residual aliasing energy associated with k‐space undersampling for each shot is fully removed following inter‐shot phase correction in the refocusing reconstruction [54]. A reduced FOV approach is particularly beneficial when in‐plane phase encoding in the direction of the wider section of the body (right → left in prostate imaging) is desired for increased robustness to breathing‐related motion artifacts from the pelvic wall.

The delay Ͳ in this sequence is chosen to be long enough to allow for sufficient available longitudinal magnetization prior to each tip‐down pulse; however, there is no requirement for this delay to allow full longitudinal magnetization recovery. Such a premise would typically entail the use of either Bloch or Extended Phase Graph simulations [31, 71] combined with dictionary matching to address T_1_‐related effects in relaxometry mapping. However, in the context of the proposed approach, a simple mono‐exponential fitting can be used instead because of two key aspects. The first aspect is the use of the magnitude stabilizers, which spoil all prepared transverse magnetization before the tip‐up pulse, meaning that the net longitudinal magnetization is zero immediately after the tip‐up pulse of each preparation. The second aspect is that the steady state magnetization will therefore depend only on the T_1_ recovery between the tip‐up pulse and the following tip‐down pulse. This explains why we keep the phase navigator pulses as dummy RF pulses for the T_2_‐prepared shots, and why the delay Ͳ was kept constant in the sequence. This delay was also kept constant in the work by Ma et al. [27], but it was assumed to be long enough for full magnetization recovery. However, since Ͳ is not long enough to allow for full T_1_ relaxation of prostatic fluids, partial volume effects may have affected T_2_ and ADC estimates [72]. It is worth noting that the magnitude stabilizer rewinder gradients spoil the regrowing unprepared inner‐volume magnetization [29, 73] which is dependent on B_1_ inhomogeneities affecting the tip‐down and tip‐up pulses. The unwanted contribution of the unprepared magnetization to the signal can be addressed in dictionary matching techniques only if transmit field inhomogeneities are taken into account in dictionary simulation and matching, and this may require a separate calibration scan. B_1_ inhomogeneities may also affect the tip up pulse in the proposed sequence, leading to imperfect saturation of the unprepared magnetization. However, this is mitigated by using a twice‐refocused preparation [74], in combination with a delay Ͳ significantly longer than diffusion or T_2_ preparation durations.

The proposed sequence enables distortion‐free imaging of the prostate in a single scan. The lack of geometric fidelity due to image distortion leads to difficulties in cancer detection and image interpretation [75] and hampers the alignment between the acquired diffusion images and other contrasts such as T_2_‐weighting. Geometric distortions can also lead to signal variations (such as signal pile up) in diffusion‐weighted images, which may affect the accuracy of the estimated ADC values [76]. The quantitative maps obtained using the proposed sequence are natively co‐registered, which facilitates analysis of spatial variations in the quantitative biomarkers. The single scan approach allows to avoid anatomical mismatch between diffusion and T_2_ data, which is one of the assessment criteria in the image quality scoring framework PI‐QUAL v2 [77] for clinical prostate MRI, where T_2_‐weighted and diffusion‐weighted scans are planned separately with a potential lack of planning consistency.

MR Fingerprinting [35] and MR multitasking [27] were also proposed as potential methods for quantifying ADC and T_2_ values within one scan. However, both approaches rely on complex, time‐consuming reconstruction algorithms. Ma et al. [27] developed a 3D gradient echo (GRE) sequence with diffusion and T_2_ preparations. Their approach is able to acquire long imaging shots because it reconstructs an image at each timepoint of the shot, hence circumventing PSF blurring. However, this also means that an additional encoding is implicitly performed in the temporal dimension, which in theory would lead to longer scan times. This is mitigated using high acceleration factors, incoherent sampling, and a low‐rank tensor reconstruction. In contrast, the approach we propose shortens imaging shots using a reduced FOV technique. In addition, shot‐specific phase was estimated in the reconstruction as an additional parameter, while the proposed approach uses an explicit phase navigator, which enables a fast and straightforward phase correction. Their approach achieves T_2_ preparation with the shortest duration possible by using a single adiabatic refocusing pulse in all T_2_ preparations. However, standard adiabatic full passage pulses have a nonlinear phase profile and may lead to incomplete refocusing due to off‐resonance [78] which is why a pair of adiabatic pulses is typically used for refocusing [53]. We therefore proposed a different approach based on a reduced T_2_ preparation to achieve the minimal T_2_ preparation duration without refocusing pulses, combined with a GRE readout with an echo time chosen close to t_T2P‐min_, while keeping a pair of adiabatic refocusing pulses in the preparations with longer durations.

Conventional T_2_‐prepared sequences [31, 73, 79, 80] acquire one image without any T_2_ preparation. However, this is not feasible in combination with the reduced FOV approach which is based on a modified preparation module. Instead, a reduced T_2_ preparation was used, introducing a small degree of T_2_* weighting. Therefore, the same delay t_T2P‐min_ was added between the second refocusing and the tip‐up pulse in the other preparation modules. It is worth noting that removing the adiabatic refocusing pulses in the reduced preparation module may have led to a change in magnetization transfer effects compared to a preparation module where these pulses are included, potentially impacting the accuracy of T_2_ mapping. A potential solution to maintain the same magnetization transfer effects would be to apply the adiabatic pulses prior to the tip‐down pulse of each reduced preparation module. They would then act as an inversion‐reinversion module, but the efficacy of such an approach may be sensitive to imperfect inversion profiles. The meSE reference sequence used in this study is also sensitive to magnetization transfer effects, as demonstrated in the work by Radunsky et al. [81].

### 4.3 Limitations

Compared to EPI‐based techniques for DWI or meSE sequences for T_2_ mapping, the proposed method enables combined ADC and T_2_ mapping in a single distortion‐free scan but suffers from low SNR efficiency due to the inherent 50% SNR penalty of stimulated echoes [48] and the delays required for longitudinal magnetization recovery between shots. This low SNR efficiency and the resulting wider limits of agreement in the estimated quantitative parameters, consistent with stimulated echo preparations, are the downside of the geometric fidelity of the proposed sequence. The reduced FOV approach was used to shorten the shots but has an associated SNR penalty compared to a fully sampled acquisition due to the acquisition of fewer phase encoding lines, although it does not suffer from g‐factor SNR penalty in parallel imaging reconstruction. The low SNR efficiency motivated the use of a moderate in‐plane spatial resolution, but within the range recommended in PI‐RADS [6] for DWI (≤ 2.5 mm). Higher spatial resolution could be achieved using self‐supervised deep learning for prostate DWI super‐resolution [82].

We did not use parallel imaging acceleration neither in the phase encoding direction nor in the partition encoding direction because of insufficient coil profile variations. However, higher spatial resolution or acceleration factors in the partition encoding direction may be achieved using deep learning‐based reconstruction methods [83, 84]. Deep‐learning [85, 86] or multidimensional, multiscale image denoising techniques [87] could also be used to mitigate the SNR penalty. Furthermore, redundancies between multiple contrasts [88, 89, 90, 91] could be leveraged to achieve 3D scans with isotropic resolution within clinically feasible scan time and reduce the likelihood of prostate motion [92] during the scan.

The current implementation of our method acquires a 2D phase navigator after the GRE imaging readout of each diffusion‐prepared shot. Since T_2_‐prepared shots do not suffer from the inter‐shot phase inconsistencies due to motion in the presence of strong diffusion‐sensitizing gradients, phase correction for these shots is not required, and phase navigator pulses are used as dummy RF pulses instead. However, these pulses could be used in a more efficient manner, for example to acquire additional k‐space lines and increase the spatial resolution of the T_2_ map.

In this study, the slice coverage was chosen to cover the prostate only, leading to a maximum coverage of 56 mm in the slice direction for the largest prostate scanned in our healthy volunteer cohort. This is because the proposed 2D phase correction strategy can be compromised when additional organs such as the bladder are captured in the FOV and projected in the 2D phase navigator images. 3D phase navigator techniques [24, 28] may be beneficial in this context.

This study has several limitations. In our phantom experiments, we investigated T_2_ mapping only, since ADC accuracy of the diffusion‐prepared sequence that we extended has already been demonstrated [30]. Furthermore, in our in vivo experiments, we used the meSE (echoes 2–6) T_2_ maps as a reference since using seSE acquisition was not possible due to infeasible long scan times. An alternative approach is to use Bloch simulations of the meSE sequence to account for imperfect refocusing pulses, slice profiles, and B_1_ inhomogeneities [62, 93]. However, this approach would require a full sequence simulation and an additional scan for mapping transmit field inhomogeneity. Finally, we acquired in vivo data in healthy volunteers only and showed only one case of a prostatic lesion. A study in patients with suspected prostate cancer is required to demonstrate any potential benefit of this sequence for lesion characterization, bi‐parametric prostate examinations, or screening purposes and to investigate slab coverage limitations due to 2D phase navigation.

## 5 Conclusion

This work proposes a 3D reduced FOV sequence for combined, distortion‐free ADC and T_2_ mapping in the prostate, enabling the use of simple mono‐exponential fitting to calculate ADC and T_2_ maps. T_2_ values in phantom and ADC values in vivo were not significantly different from the reference acquisition. An expert reader study confirmed that the method is robust to geometric distortions compared to EPI‐based diffusion‐weighted imaging techniques.

## Funding

The authors have nothing to report.

## Conflicts of Interest

Y.O. is an employee of Siemens Healthineers and his tuition fees are covered by Siemens Healthineers. O.D., S.M., and R.T.‐T. are employees of Siemens Healthineers.

## Supporting information


**Table S1:** Sequence parameters for phantom studies. Please note that for the proposed sequence T2 and diffusion preparation durations are the main factor behind the amount of T2 weighting while it is TE for all the spin echo sequences used in this study (ssEPI, rFOV ssEPI, meSE, and TSE).
**Figure S1:** Through‐slice animation of b800 trace‐weighted images for the proposed acquisition, with and without 2D phase correction. Reference ssEPI images are also shown.
**Figure S2:** Comparison of diffusion‐weighted images, including a calculated b1400 trace‐weighted image, and ADC maps in one representative volunteer. Diffusion data were acquired using a single‐shot EPI, reduced FOV single‐shot EPI, and the proposed sequence. b800 and b1400 (calculated) trace‐weighted images are displayed with increased brightness (×2).

## Data Availability

The data that support the findings of this study are available on request from the corresponding author. The data are not publicly available due to privacy or ethical restrictions.
