# Supplementary material for: Combined ADC and T2 Mapping in the Prostate Using a 3D Reduced FOV Sequence
Source: Magn Reson Med. 2026 Jul 14;96(5):2292–308. doi: 10.1002/mrm.70510 (PMC13527244; doi:10.1002/mrm.70510)
Supplement: Supplementary file 1 [file MRM-96-2292-s001.docx]

SUPPLEMENTARY MATERIAL

Table S1: Sequence parameters for phantom studies. Please note that for the proposed sequence T2 and diffusion preparation durations are the main factor behind the amount of T2 weighting while it is TE for all the spin echo sequences used in this study (ssEPI, rFOV ssEPI, meSE, and TSE).

|  | Proposed | meSE | seSE |
| --- | --- | --- | --- |
| TE (spin echo) [ms] | - | 20, 40, 60, 80, 100, 120 | |
| TE (gradient echo) [ms] | 3 | - | |
| TR [ms] | 9710 – 9790* | 10000 | |
| FOV (read x phase)  [mm x mm] | 300 x 75 | 300 x 300 | |
| Number of slices | 48 | 1 | |
| Phase oversampling  [%] | 10 | None | |
| Receiver bandwidth  [Hz/Px] | 490 | 200 | 130 |
| Excitation flip angle [˚] | 10 | 90 | |
| Acquisition time [h:min:s] | 00:23:24 | 00:21:10 | 02:07:00 |

**Abbreviations**: meSE, multi-echo spin-echo; seSE, single-echo spin-echo;

*Variable for different preparation modules

Figure S1: Through-slice animation of b800 trace-weighted images for the proposed acquisition, with and without 2D phase correction. Reference ssEPI images are also shown.

Figure S2: Comparison of diffusion-weighted images, including a calculated b1400 trace-weighted image, and ADC maps in one representative volunteer. Diffusion data were acquired using a single-shot EPI, reduced FOV single-shot EPI, and the proposed sequence. b800 and b1400 (calculated) trace-weighted images are displayed with increased brightness (x2).
